# Elective induction versus expectant management for suspected large-for-gestational-age fetuses: a systematic review and meta-analysis

**DOI:** 10.1186/s12884-026-08787-x

**Published:** 2026-02-20

**Authors:** Abd-alrahman Al-Qudah, Mohammad Aleidi, Husam Alshebelat, Mohammad Al-Hanaktah, Reham Albadaineh, Edward Mullins

**Affiliations:** 1 Royal Jordanian Medical Services, Amman, Jordan; 2 Ministry of Health, Amman, Jordan; 3 Faculty of Medicine, University of Jordan, Amman, Jordan; 4 Department of Metabolism, Digestion and Reproduction & The George Institute for Global Health, Imperial College London, London, UK; 5 Honorary Consultant, Imperial College Healthcare NHS Trust, London, UK

**Keywords:** Induction of labour, Macrosomia, Shoulder dystocia, Obstetrics, Meta-analysis

## Abstract

**Background:**

Suspected large-for-gestational-age (LGA) fetuses present a clinical dilemma: early induction may reduce birth trauma but raise intervention risks. Previous reviews lacked recent data.

**Objectives:**

To assess whether elective induction at 37–39 weeks reduces adverse maternal and neonatal outcomes compared with expectant management in pregnancies with suspected LGA fetuses.

**Methods:**

We systematically searched PubMed, Cochrane Library, Scopus, Web of Science, and ClinicalTrials.gov through May 2025, with no language restrictions. We included randomised controlled trials (RCTs) comparing elective induction (37–39 weeks) with expectant management in singleton pregnancies with suspected (LGA) fetuses. Two reviewers independently screened studies, extracted data, and assessed the risk of bias using RoB 2.0. Outcomes were pooled using fixed- or random-effects meta-analysis, and the certainty of evidence was evaluated using the GRADE framework.

**Main results:**

Three RCTs (*n* = 3,984) met the inclusion criteria. Induction significantly reduced shoulder dystocia (RR 0.65, 95% CI 0.46–0.91), caesarean birth (RR 0.87, 95% CI 0.79–0.95), and increased spontaneous vaginal birth (RR 1.12, 95% CI 1.06–1.19). No differences were seen in instrumental delivery, severe perineal trauma, or perinatal death. Induction lowered mean birthweight (–177 g, 95% CI − 279 to − 76) but was associated with increased neonatal phototherapy (RR 1.63, 95% CI 1.19–2.23). Certainty of evidence was moderate for most primary outcomes.

**Conclusions:**

For suspected LGA fetuses, induction around 38 weeks reduces birth trauma and caesarean risk without increasing major maternal or neonatal morbidity. Clinical discussions should weigh these benefits against patient preferences and contextual factors.

**Supplementary Information:**

The online version contains supplementary material available at 10.1186/s12884-026-08787-x.

## Introduction

### Induction of labour versus expectant management in suspected macrosomia

Fetal macrosomia (variously defined as birthweight ≥ 4000–4500 g) and large-for-gestational-age (LGA) (variously defined as estimated fetal weight > 90th–95th percentile) affect a significant proportion of pregnancies [1, 2]. The prevalence of suspected LGA has risen over time, driven by increasing maternal obesity and diabetes [3, 4]. 

LGA babies carry a markedly higher risk of adverse perinatal outcomes: LGA fetuses are at increased risk of shoulder dystocia (an obstetric emergency where the fetal shoulder impinges on the maternal pelvis) and associated injuries to the brain and brachial plexus. Shoulder dystocia complicates 0.3–3% of vaginal deliveries; the risk rises significantly with fetal macrosomia (odds ratio ~ 16 for macrosomic fetuses) [5–7]. Large-for-gestational-age infants also have increased rates of brachial plexus injury, long bone fractures, and hypoxic brain injury at birth [8]. For mothers, delivering a large baby is associated with higher rates of prolonged labor, severe perineal trauma (third- or fourth-degree lacerations), postpartum hemorrhage, and emergency caesarean delivery [9]. These serious maternal and perinatal morbidities underscore the clinical relevance of suspected macrosomia as a concern for women, obstetricians, midwives and neonatologists. They are therefore likely to lead to higher rates of obstetric interventions.

### Clinical definitions and rationale

Definitions of abnormally large fetal size vary. The American College of Obstetricians and Gynecologists (ACOG) notes that “macrosomia” usually refers to an absolute weight (historically > 4000 g or > 4500 g), regardless of gestational age [1]. By contrast, “large-for-gestational-age” is a percentile-based definition (often the 90th or 95th percentile for gestational age). For example, NICE defines suspected LGA as an estimated fetal weight above the 95th percentile at ≥ 36 weeks’ gestation [2]. Fetal weight estimation has limitations, but identification of a suspected large fetus is clinically important because it triggers consideration of aetiology and intervention to mitigate associated risks [9]. 

Given the potential for harm to pregnancies with large babies and the risks of interventions, the management of suspected macrosomia is clinically debated. One strategy is elective induction of labour in the late third trimester to deliver the large fetus earlier, to reduce birth weight and related trauma [2]. Alternatively, expectant management (awaiting spontaneous labor or traditional timing of induction)or planned caesarean section can be considered. The decision to proceed with early induction weighs the potential to reduce fetal size and shoulder dystocia risk against the possibility of increasing operative delivery and perineal trauma.

### Guidelines and current practice

Clinical guidelines reflect the uncertainty in this area of obstetric practice. ACOG’s recent bulletin states that suspected macrosomia alone is not an indication for induction before 39 weeks, because evidence was considered insufficient that earlier delivery reduces harm [1]. ACOG instead focuses on the optimal timing and mode of delivery based on estimated weight thresholds (for example, considering caesarean delivery if the estimated fetal weight is ≥ 5,000 g in non-diabetic women or ≥ 4,500 g in diabetic women) [1]. In contrast, NICE (UK) guidelines advocate individualized discussion. NICE recommends that women with suspected LGA be counseled on options (expectant management, induction, or caesarean) and specific outcome differences [2]. Notably, NICE cites evidence that induction reduces shoulder dystocia risk but increases the risk of severe perineal tears while showing no apparent difference in perinatal death, brachial plexus injury, or emergency caesarean rate between strategies [2]. Thus, NICE acknowledges trade-offs and emphasizes shared decision-making. In practice, local policies vary: some clinicians offer early induction around 37–39 weeks for suspected macrosomia, while others await labor, reflecting differing interpretations of the evidence.

### Evidence from trials and reviews

To date, trials of induction versus expectant management in suspected macrosomia have been relatively few and small. The most recent Cochrane review (2023) identified only four randomized trials (total n1190) comparing induction near-term versus expectant management for macrosomia [10]. That meta-analysis found no statistically significant difference in caesarean delivery (RR ≈ 0.91, 95%CI 0.76–1.09). However, induction was associated with significantly lower rates of shoulder dystocia (RR ≈ 0.60, 95%CI 0.37–0.98) and neonatal fractures (RR ≈ 0.20, 95%CI 0.05–0.79). There was no apparent effect on brachial plexus injuries (an uncommon outcome) or neonatal acid-base status. Induction also yielded a modest reduction in mean birth weight. Overall, the Cochrane review authors concluded that induction of labor for suspected macrosomia “results in a lower mean birthweight, and fewer birth fractures and shoulder dystocia,”. However, that evidence was limited by small sample sizes and lack of blinding. They explicitly noted the need for further large trials to refine the balance of risks and benefits. They noted the increased risk of phototherapy for babies whose mothers had induction of labour.

Previous systematic reviews (Cochrane review,2015) judged the existing evidence too imprecise to make firm practice recommendations.

In summary, previous evidence syntheses suggested potential neonatal benefits from induction (including fewer shoulder dystocia events and fractures) without clear maternal benefit, but confidence intervals were wide and the effects on caesarean section risk remained unclear.

### Rationale for an updated systematic review

Several developments justify a new comprehensive review. First, very recently, a large multicenter trial, the “Big Baby” trial, *n* = 2893, has been published (Lancet 2025) [11], dramatically expanding the randomized evidence on induction for suspected LGA. This study showed no benefit of IOL for shoulder dystocia by ITT analysis, but a benefit was seen by per-protocol analysis. Second, many guideline bodies (e.g., NICE) have highlighted macrosomia as a priority research area precisely because current evidence is sparse and practice varies [2, 11]. NICE explicitly encourages recruitment into trials on induction for macrosomia [2]. Third, no prior review has yet integrated this new large trial. Finally, accurate fetal weight estimation remains imperfect, so even women without actual macrosomic babies may face interventions; this underscores the need for precise pooled risk estimates to inform consent. An updated systematic review and meta-analysis, incorporating all recent trials and using comprehensive methodology (e.g., GRADE assessment), will therefore clarify the balance of maternal and perinatal outcomes with induction versus expectant management. Such evidence is crucial to resolve current uncertainties, directly inform clinical guidelines (including for women with and without diabetes), and guide shared decision-making for women facing a suspected large baby.

## Methods

We conducted this systematic review and meta-analysis in accordance with the Cochrane Handbook for Systematic Reviews of Interventions (Version 6.4). We reported it following PRISMA 2020 reporting guidelines. The completed PRISMA 2020 checklist is provided in Supplementary Appendix 1. The protocol was registered prospectively in PROSPERO (CRD420251045480); no amendments were made after registration [12]. 

### Search strategy and selection criteria

We developed a comprehensive search strategy using both controlled vocabulary (MeSH, Emtree) and free-text terms for labor induction, expectant management, and macrosomia/large-for-gestational-age. Six electronic databases—MEDLINE (Ovid), Embase (Ovid), CENTRAL, CINAHL, ClinicalTrials.gov, and the WHO ICTRP—were searched from database inception to 3 May 2025, with no language, date, or geographic limits.

For this review, we restricted inclusion to peer-reviewed, published randomized controlled trials. Data from unpublished or grey literature sources were not included in the primary analyses, as eligibility assessment was based on information provided in peer-reviewed publications. We did not contact the original trial investigators for additional or unpublished data. Reference lists of the included trials and relevant systematic reviews were screened for additional published RCTs, but none beyond our electronic search results met the eligibility criteria.

To explore the potential impact of excluding unpublished trials in a field with relatively few RCTs, we conducted a sensitivity analysis incorporating unpublished data extracted from previous systematic reviews. These data were independently collected from previous reviews and are presented in Supplementary Appendix 2.

Eligible studies were parallel-group randomized controlled trials comparing elective induction of labour at ≥ 37 weeks with expectant management in singleton, cephalic pregnancies suspected to be LGA (estimated fetal weight ≥ 4000 g or > 90ᵗʰ–95ᵗʰ centile). Trials enrolling women with pre-existing diabetes were eligible, whereas studies involving multiple pregnancies, non-cephalic presentation, quasi-randomization, or observational designs were excluded. Two reviewers independently screened titles/abstracts and full texts in Rayyan.ai [13]. Disagreements were resolved by discussion or, when necessary, consultation with a third reviewer. Reasons for full-text exclusion were recorded, and the selection process was summarised in a PRISMA flow diagram.

## Data collection and analysis

Data were extracted independently by two reviewers using a piloted form capturing study design, setting, participant characteristics, induction protocol, comparator details, and outcome data. In accordance with our PROSPERO-registered protocol (CRD420251045480) and the COMET/WHO core outcome set for induction of labour [14]. Prioritising outcomes reported with standard definitions across trials, allowing valid pooling. Prespecified primary outcomes were:

1. Shoulder dystocia (any maneuver beyond routine traction)
2. Caesarean section (any indication)
3. Instrumental vaginal delivery (vacuum or forceps)
4. Brachial plexus injury (Erb’s/Klumpke’s palsy diagnosed ≤ 28 days postpartum)

Secondary outcomes included mean birth weight, spontaneous vaginal birth, severe perineal trauma, major postpartum haemorrhage, neonatal fractures, NICU admission, need for phototherapy, and perinatal mortality.

The risk of bias for each primary outcome in every trial was assessed independently with the Cochrane RoB 2 tool; judgments (“low risk”, “some concerns”, “high risk”) were reconciled by consensus [15]. 

All quantitative syntheses were run in R, and the pooled estimates, forest plots, and heterogeneity statistics were independently cross-checked in Review Manager (RevMan) version 5.4 to confirm numerical concordance [16, 17]. For dichotomous outcomes, we calculated risk ratios (RR) with 95% confidence intervals (CI) and pooled them using the Mantel–Haenszel inverse-variance method [18]. Continuous outcomes were summarised as mean differences (MD). Statistical heterogeneity was assessed with the χ² test (*P* < 0.10) and quantified with *I²*. A fixed-effect model was used when *I²* ≤ 50%; otherwise, reasons for heterogeneity were explored and a random-effects model was applied [18]. Publication bias for the primary outcomes was not formally assessed with Egger’s test or contour-enhanced funnel plots because fewer than ten trials were available, rendering such tests unreliable.

Finally, we rated the certainty of evidence for each primary outcome using GRADE, downgrading for risk of bias, inconsistency, indirectness, imprecision, and publication bias as appropriate [19]. Full GRADE ratings and detailed downgrading justifications for all outcomes are provided in Table S1. To further support confidence in the results, we also applied the TRACT checklist to assess the trustworthiness of the included trials [20]. 

## Results

### Search results

**Fig. 1:**
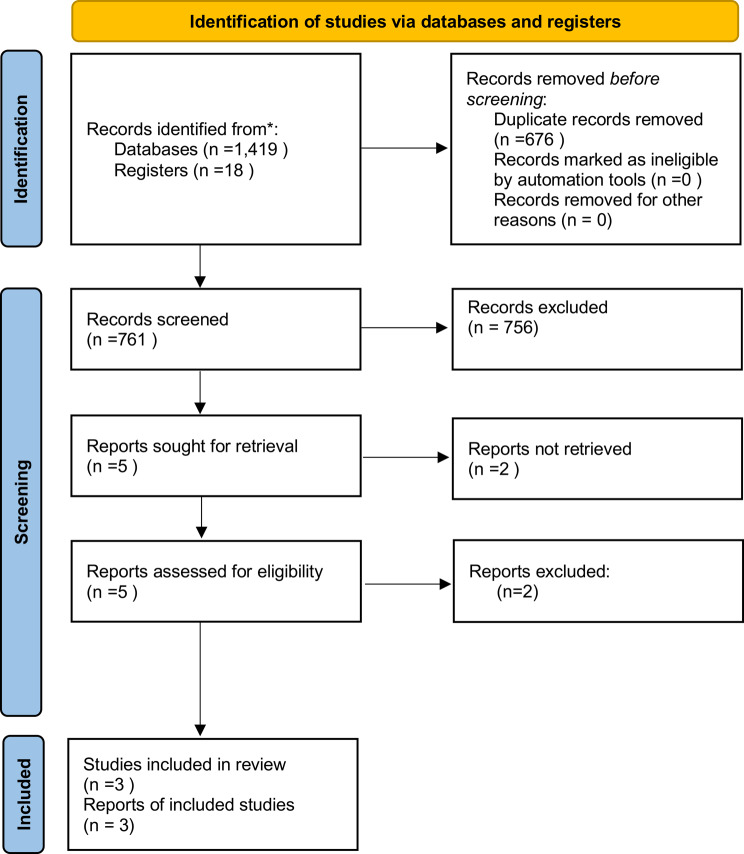
PRISMA 2020 study‑selection flow‑chart. Searches of MEDLINE, Embase, CINAHL, and CENTRAL (1 419 records) plus trial registers (18 records) produced 1 437 citations. After the removal of 676 duplicates, 761 unique titles/abstracts were screened; 756 were excluded. Five full‑text reports were assessed, two were excluded (one non‑RCT, one wrong population) and three randomized controlled trials (RCTs) were therefore included in the review and meta‑analysis

We retrieved 1 419 records through electronic searches of the Cochrane Library, PubMed, Scopus, Web of Science, and ClinicalTrials.gov up to 3 May 2025. After removing 676 duplicates, we screened 761 unique titles and abstracts. We excluded 756 records that did not meet our eligibility criteria (for example, non-randomized designs or irrelevant populations). Five full-text articles arose from the database search. Back-citation screening uncovered two additional unpublished trials—Libby 1998 and Tey 1995—cited in earlier reviews; as neither was available in full text, both were excluded at this stage but later considered in sensitivity analyses using data extracted from prior reviews (Supplementary Appendix 2). Consequently, five articles underwent full-text assessment. Two of these were not randomised controlled trials, leaving three published RCTs for qualitative and quantitative synthesis. Fig. 1 presents the PRISMA 2020 flow diagram; no automation tools affected the final counts.

### Characteristics and quality of included studies

Three parallel group randomized controlled trials met the eligibility criteria: Boulvain et al. (France, Switzerland, Belgium), Gonen et al. (Israel), and the UK Big Baby trial Gardosi et al. [11, 21, 22]. All were open‑label designs powered to detect differences in delivery morbidity, although sample size varied markedly (*n* = 818, 273, and 2 893, respectively). Randomization was judged low risk in all three trials (ROB-2 D1). Because the interventions were necessarily open-label, every study carried “some concerns” for deviations from intended interventions (D2). Missing-data bias was low (D3): attrition was < 2% and balanced between arms throughout. Outcome measurement was low risk in Boulvain 2015 and Gardosi 2025, but only “some concerns” in Gonen 1997 because assessor blinding was not documented (D4). Selective-reporting bias was low across the board (D5). Overall, ROB-2 ratings were “some concerns” for Boulvain and Gardosi, and “high risk” for Gonen, with the chief limitations being lack of blinding and the unclear outcome-assessment procedures in the earlier trial. A visual summary of the risk-of-bias assessments is provided in Figure S1. Trustworthiness was further assessed using the TRACT checklist. The UK Big Baby trial (Gardosi 2025) was rated as having “no concerns” across all seven domains. Boulvain 2015 had “some concerns” for governance but was otherwise fully reassuring. Gonen 1997 raised “some concerns” in three domains—governance, timeframe, and baseline balance—reflecting its earlier conduct and limited reporting. Overall, TRACT judgments were “no concerns” for Gardosi, and “some concerns” for both Boulvain and Gonen. Full domain-level ratings are provided in Supplementary Table S2. Key methodological and clinical characteristics of the three trials are summarised in Table 1.

**Table 1:** Characteristics of the randomized controlled trials included in the review

Summary of Included Randomised Controlled Trials									
Study (year)	Setting/ centres	Sample size (Induction / Expectant)	GA at randomisation (weeks)	LGA / macrosomia definition	Induction protocol	Comparator (expectant-management policy)	Key outcomes reported	RoB 2 overall rating	Overall TRACT
Boulvain et al. 2015 [10]	France, Switzerland and Belgium; 19 hospitals	409 / 413	36 + 0–38 + 0	EFW > 95ᵗʰ centile	Vaginal PGE₂ (3 mg) → amniotomy ± oxytocin	Await spontaneous labour; routine induction ≥ 41 weeks	CS, instrumental delivery, shoulder dystocia, birth‑weight, neonatal trauma	Some concerns	Some concerns
Gardosi “Big Baby” 2025 [11]	UK; 106 NHS units	1 447 / 1 446	35 + 0–38 + 0	EFW ≥ 90ᵗʰ centile (GROW customised)	Standard practice of the participating hospital	Spontaneous labour or induction ≥ 38 + 4 weeks	CS, shoulder dystocia, maternal and neonatal morbidity	Some concerns	No concerns
Gonen et al. 1997 [21]	Israel; single centre	134 / 139	38 + 0–38 + 6	EFW ≥ 4 000 g and < 4500 g	PGE₂ or oxytocin	Expectant until labour or ≥ 42 weeks	CS, shoulder dystocia, neonatal birth injury	High concerns	Some concerns

Study setting, sample size, gestational‑age window at randomization, LGA/macrosomia definition, induction regimen, comparator policy, reported maternal/neonatal outcomes, and overall RoB 2 judgment

*Abbreviations*: *CS* Caesarean section, *EFW *Estimated fetal weight, *GA *Gestational age, *GROW *Gestation Related Optimal Weight, *NICU *Neonatal intensive‑care unit, *PGE₂* Dinoprostone, *PPH *Post‑partum haemorrhage, *RoB 2 *Cochrane Risk‑of‑Bias 2 tool

### Gestational age and intervention protocols

Gonen 1997 randomized women from 38 weeks with an ultrasonographic fetal weight estimate of 4 000–4 500 g. Induction (oxytocin or prostaglandin according to Bishop score) commenced immediately; controls received post‑date surveillance (twice‑weekly testing) and were induced at 42 weeks if undelivered.

Boulvain 2015 induced labour within 72 h of randomization between 37 + 0 and 38 + 6 weeks; methods (prostaglandin E₂, misoprostol, or oxytocin) followed local policy. Expectant care continued until spontaneous labor or another clinical indication, with routine post-date induction after 41 weeks.

Big Baby 2025 offered induction between 38 + 0 and 38 + 4 weeks (protocol target ≤ 38 + 4) using the site’s standard cervical‑ripening strategy. Women receiving standard care were counselled to avoid elective delivery before 38 + 4 weeks unless clinically indicated.

Across trials, induction was consistently timed in the early‑term window, with controls managed expectantly to 39–41 weeks.

### Participant characteristics

Baseline maternal characteristics were broadly comparable within and across studies (Table 2). Mean maternal age ranged from 28.8 years (Big Baby) to 30.8 years (Gonen). Nulliparity varied: 56.7% in Big Baby, 49% in Boulvain, and 30% in Gonen. In Boulvain the mean pre‑pregnancy BMI was 26 kg/m²; in Big Baby, 62% of women were overweight or obese (BMI ≥ 25 kg/m²). Diet‑controlled gestational diabetes affected 4–5% of participants and 10–11% in Boulvain. While smoking prevalence was ~ 10% in Big Baby, and was unreported in the other studies. Only Gardosi 2025 included women with a previous caesarean Sect. (2.4%) (Table 2).

**Table 2:** Baseline characteristics and screening populations of the included randomized controlled trials

Characteristic	Boulvain et al. 2015 [10]	Gardosi (Big Baby) 2025 [11]	Gonen et al. 1997 [21]
Mean Maternal Age	~ 30 years	28.8 years	30.8 years
Nulliparity Rate	49%	57%	30%
BMI / Obesity	Mean BMI: 26 kg/m²	62% BMI ≥ 25 kg/m²	Not reported
Diabetes Status	10–11% Diet-controlled GDM	4–5% Diet-controlled GDM	Excluded
Previous C-Section	Excluded	2.4%	Excluded
Previous Macrosomia	31%	16–17%	Not reported
History of Shoulder Dystocia	Not reported	2%	Not reported
Smoking Status	Not reported	~ 10%	Not reported
Screening Rationale	Universal screening at 37–38 weeks	Routine growth monitoring	Suspected weight at 38 weeks
LGA Definition	EFW > 95th centile	Customized EFW > 90th centile	EFW 4,000–4,499 g

Maternal demographic characteristics, obstetric history, metabolic status, screening rationale, and definitions used to identify suspected large-for-gestational-age fetuses across trials. Variables include maternal age, nulliparity, body mass index or overweight/obesity prevalence, gestational diabetes status, prior caesarean section, previous macrosomia or shoulder dystocia, smoking status, and fetal-size screening criteria

*Abbreviations*: *BMI* Body mass index, *CS *Caesarean section, *EFW *Estimated fetal weight, *GA* Gestational age, *GDM *Gestational diabetes mellitus, *LGA *Large-for-gestational-age

Eligibility thresholds for fetal size differed: Boulvain used an estimated fetal weight > 95th percentile (≈ 3 700–3 900 g at 37–38 weeks); Gonen used EFW (4 000–4 500 g); Big Baby used customized EFW> 90th percentile between 35 weeks and 0 days (35 + 0 weeks) and 38 + 0 weeks gestation. Sonographic estimated fetal weight at randomization averaged 3,964 g (Boulvain) and 4 160 g (Gonen). Recruitment occurred between 35 + 0 and 38 + 0 weeks in Big Baby, 36 + 0 to 38 + 0 weeks in Boulvain, and after 38 weeks in Gonen.

Collectively, the trials enrolled predominantly healthy women without diabetes with suspected LGA fetuses in late term; ethnicity was reported only in the UK study. These similarities support the appropriateness of pooling data, acknowledging that differences in fetal‑weight thresholds and national practices may contribute to heterogeneity. A graphical summary of the study design, core findings, and timeline of included trials is presented in Figure S2.

### Perinatal injury to the baby

**Fig. 2:**
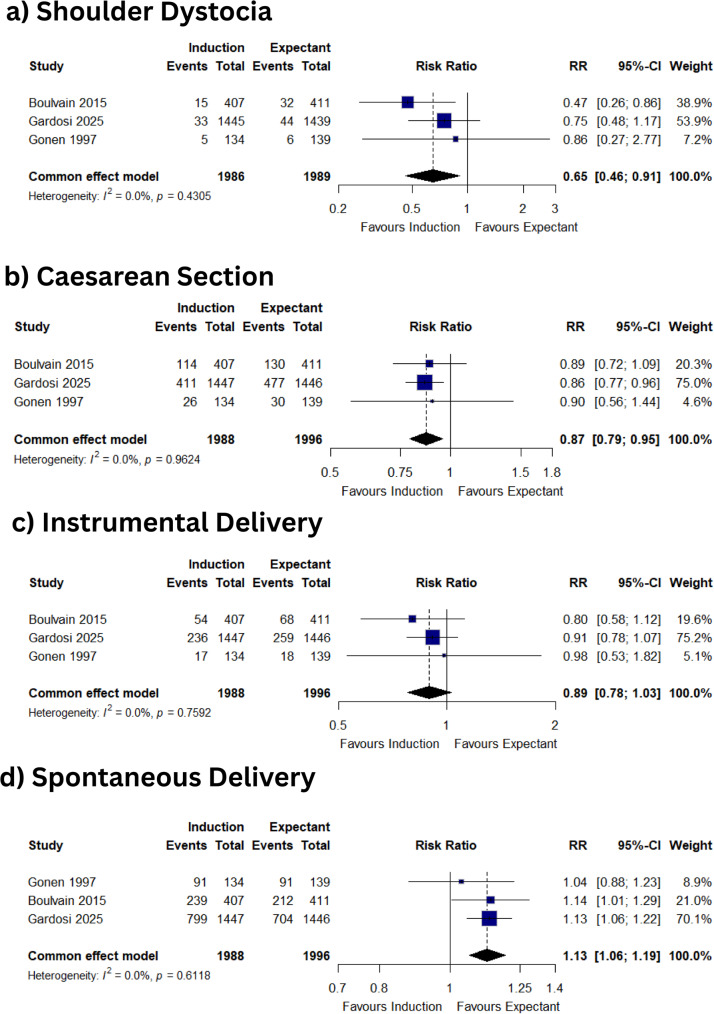
Pooled effects of labour‑induction versus expectant management for suspected large‑for‑gestational‑age (LGA) fetuses. Forest plots show common-effect (fixed) estimates for (**A**) shoulder dystocia, (**B**) Caesarean section, (**C**) instrumental vaginal delivery, and (**D**) spontaneous vaginal delivery. Squares represent study-specific risk ratios weighted by inverse variance; horizontal lines denote 95 % confidence intervals (CIs). Diamonds indicate pooled estimates, and the vertical line marks no effect (RR = 1·0). Heterogeneity was negligible for all outcomes (I^²^ = 0 %)

Induction of labour between around 38 weeks reduces shoulder dystocia by one-third (RR 0.65, 95% CI 0.46–0.91; I² = 0%) (Fig. 2A). Brachial plexus injury and birth fracture are uncommon; estimates suggest possible benefit but are imprecise: RR 1.01 (0.27–3.75) and RR 0.20 (0.05–0.79), respectively (Figures S4A, S4C).

### Maternal perinatal outcomes

Among 3,984 women, induction reduces caesarean birth by 13% (RR 0.87, 0.79–0.95; I² = 0%) (Fig. 2B), without increasing instrumental vaginal birth (RR 0.89, 0.78–1.03) (Fig. 2C). Spontaneous vaginal birth is slightly more frequent (RR 1.13, 1.06–1.19) (Fig. 2D). Obstetric anal sphincter injury is infrequent and remains uncertain (RR 1.10, 0.72–1.69) (Figure S4B). Major primary Postpartum haemorrhage (> 1000 ml blood loss), reported in two trials, shows no clear effect (RR 0.89, 0.75–1.06) (Figure S4E).

### Early neonatal adaptation and mortality

Induction leads to lower birth weight (MD − 177 g, − 279 to − 76; I² = 93%) (Figure S1A) and increases phototherapy use (RR 1.63, 1.17–2.25) (Figure S3B). No significant differences are seen for NICU admission (RR 0.94, 0.58–1.52) (Figure S3C) or Apgar < 7 at 5 min (RR 1.61, 0.90–2.89) (Figure S3D). Perinatal death is rare (1 in 3,741); the pooled estimate is uninformative (RR 1.00, 0.06–15.96) (Figure S4D). An overview of pooled effect estimates and GRADE certainty ratings for all prespecified outcomes is provided in Table 3. 

**Table 3:** GRADE ‘Summary of findings’ table

Outcome	Risk with induction (per 1000)	Risk with expectant (per 1000)	Relative effect (95% CI)	Participants (studies)	Certainty of evidence (GRADE)
Shoulder dystocia	27	41	RR 0.65 (0.46 to 0.91)	3 975 (3 RCTs)	⨁⨁⨁◯ Moderate¹
Caesarean section	277	319	RR 0.87 (0.79 to 0.95)	3 984 (3 RCTs)	⨁⨁⨁◯ Moderate¹
Instrumental vaginal delivery	154	173	RR 0.89 (0.78 to 1.03)	3 984 (3 RCTs)	⨁⨁◯◯ Low¹ ²
Spontaneous vaginal birth	568	505	RR 1.13 (1.06 to 1.19)	3 984 (3 RCTs)	⨁⨁⨁◯ Moderate¹
Brachial plexus injury	2	2	RR 0.92 (0.11 to 7.58)	3 984 (3 RCTs)	⨁◯◯◯ Very low¹ ² ³
Fracture (any)	1	6	RR 0.20 (0.05 to 0.79)	3 984 (3 RCTs)	⨁◯◯◯ Very low¹ ² ³
Low Apgar < 7 (5 min)	16	10	RR 1.61 (0.90 to 2.89)	3 711 (2 RCTs)	⨁◯◯◯ Very low¹ ² ³
3°/4° anal sphincter tear	23	21	RR 1.1 (0.72 to 1.69)	3 711 (2 RCTs)	⨁◯◯◯ Very low¹ ² ³
Perinatal mortality	0.5	0.5	RR 1.00 (0.06 to 15.96)	3 711 (2 RCTs)	⨁◯◯◯ Very low² ³ ⁴
NICU admission	92	87	RR 0.94 (0.58 to 1.52)	3 711 (2 RCTs)	⨁◯◯◯ Very low¹ ² ³
Mean birthweight (g)	M = 3 941	M = 4 118	MD –177 g (–278 to − 75)	3 984 (3 RCTs)	⨁⨁⨁◯ Moderate¹ ⁵
Major Postpartum Haemorrhage	116	130	RR 0.89 (0.75 to 1.06)	3 711 (2 RCTs)	⨁⨁◯◯ Low¹ ²
Phototherapy	48	30	RR 1.63 (1.17 to 2.25)	3 711 (2 RCTs)	⨁⨁⨁◯ Moderate¹

Absolute risks with expectant management, corresponding risks with induction, pooled effect estimates (RR or MD) with 95 % CIs, total participants/trials, and overall GRADE certainty for each prespecified maternal or neonatal outcome. ¹downgraded one level for risk of performance bias due to open-label study design; ²downgraded one level for imprecision, defined as wide confidence intervals or few events; ³downgraded one level for very low event rates resulting in serious imprecision; ⁴downgraded one level due to rarity of the outcome leading to very wide confidence intervals spanning clinically important benefit and harm; ⁵downgraded one level for inconsistency, defined as substantial heterogeneity (I² > 75%)

Certainty assessment follows GRADE methodology. Explanations:

1. Downgraded one level for risk of performance bias (open‑label).
2. Downgraded one level for imprecision (wide confidence intervals or few events).
3. Downgraded one level for very low event rate (serious imprecision).
4. Downgraded one level for rarity of outcome leading to very wide CI spanning important benefit and harm.
5. Downgraded one level for inconsistency (I² > 75%).

### Sensitivity analyses

In sensitivity analyses that incorporated data from the older, small unpublished trials identified in previous reviews, the pooled estimates were very similar to those of the primary analyses, and no change in the direction of effect was observed. Although these trials were at higher risk of bias and lacked full methodological reporting, their inclusion did not materially change the direction or magnitude of effect estimates, suggesting that their omission from the primary analysis is unlikely to have introduced important bias (supplementary Appendix 2).

## Discussion

### Main findings

Early-term induction in suspected LGA pregnancies markedly lowers both shoulder dystocia and caesarean delivery rates, with no increase in instrumental delivery or other operative interventions and without effect on maternal adverse outcomes. Importantly, induction reduces the risk of caesarean delivery and increases the likelihood of spontaneous vaginal birth, with rates of major postpartum haemorrhage remaining comparable between groups. There is no significant increase in severe neonatal birth trauma (e.g., fractures) following induction. However, induction is associated with a higher need for neonatal phototherapy. There are no significant differences in rare outcomes such as brachial plexus palsy or perinatal death.

### Neonatal phototherapy

Induction was associated with increased neonatal phototherapy (RR 1.63, 95% CI 1.17–2.25; moderate certainty), driven by data from two trials [10, 11]. In Boulvain 2015, the highest reported bilirubin concentrations did not exceed thresholds for severe hyperbilirubinemia (≥ 350 µmol/L), while Gardosi 2025 did not report peak bilirubin levels; these episodes likely represented transient physiological immaturity due to earlier gestational age at delivery rather than pathological jaundice. Clinicians should interpret this increase cautiously—as a common, low-severity finding that rarely requires prolonged intervention—while integrating it into the overall risk–benefit profile: induction reduces shoulder dystocia and caesarean rates without elevating severe neonatal morbidity, but warrants counseling on potential short-term postnatal monitoring.

### Interpretations

Findings of this meta-analysis extend and refine the evidence base in several important ways. The magnitude and direction we observed for both shoulder dystocia and spontaneous vaginal birth are consistent with the largest previous randomized trial [10], which showed a two-thirds reduction in shoulder dystocia and no increase in caesarean delivery after induction between 37 and 39 weeks [10]. The 2023 Cochrane review, which pooled four smaller trials completed before 2016, reported a similar shoulder-dystocia benefit (RR ≈ 0.60) but no clear effect on caesarean section (RR 0.91, 95% CI 0.76–1.09) [10]. By incorporating the 2 893-woman Big Baby trial published after the Cochrane search window, this meta-analysis triples the included sample size and demonstrates a statistically significant reduction in caesarean birth (RR 0.87, 95% CI 0.79–0.95), while confirming the reduction of early term induction on the shoulder-dystocia analysed by ITT, which the Big Baby study only showed on per protocol analysis [11]. This contrasts with an earlier BJOG review by Magro-Malosso et al. which concluded that induction did not alter risk of caesarean section and that evidence for reduction in shoulder-dystocia was inconclusive [23]. 

Our results also challenge the conclusions drawn from older observational work. Sanchez-Ramos et al.’s 2002 meta-analysis of non-randomized studies suggested induction *increased* caesarean rates for women with LGA babies [24]. however, those studies were prone to selection bias (higher-risk women preferentially induced). Subsequent RCTs, including our pooled dataset reported that early-term induction reduced the risk of caesarean section when gestational age was comparable.

Guideline committees have until now favoured a cautious approach to intervention for suspected LGA. The ACOG currently discourages elective induction for suspected macrosomia before 39 + 0 weeks, citing insufficient evidence of net benefit [1]. The clearer reduction in both shoulder dystocia and caesarean section shown in this meta-analysis suggests that the stance and other clinical guidelines should be revisited.

### Strengths and limitations

This review synthesizes all available published randomized evidence on induction for suspected LGA, including the largest trials and recent high-quality studies. By focusing on published RCTs, we minimize bias compared to older observational analyses. We also assessed outcomes with GRADE methodology (the evidence on shoulder dystocia was rated moderate quality). However, limitations exist. The total number of trials is still small; one of these had a relatively small sample size. The Big Baby trial (Gardosi 2025; *n* = 2,893) contributed ~ 73% of participants (2,893/3,984), exerting substantial influence on pooled estimates. For primary outcomes, its weight was 82% for shoulder dystocia, 75% for caesarean section, and 72% for instrumental delivery (forest plots, Figs. 2, S3–2). Removing Big Baby yields consistent directions (e.g., shoulder dystocia RR 0.52, 95% CI 0.25–1.07 from Boulvain/Gonen) but wider CIs due to smaller samples. Pooled results thus largely reflect Big Baby’s findings, though consistency across trials supports robustness; readers should interpret with awareness that further large RCTs are needed for precision. Notably, only Gardosi 2025 included women with prior caesarean section (~ 3–4%), while Boulvain 2015 and Gonen 1997 explicitly excluded them. In addition, most inductions in the included trials occurred at or after 38 weeks’ gestation, which may restrict the generalizability of findings to earlier gestational ages or alternative timing strategies. There was moderate clinical heterogeneity (e.g., differences in exact gestational age at induction, methods of induction, and LGA definitions), which can dilute pooled estimates. Most trials could not blind providers or patients, introducing potential performance bias (though outcomes like shoulder dystocia are objective). The accuracy of sonographic fetal weight is limited, so many “suspected LGA” fetuses were ultimately of average size; this misclassification may bias results toward the null [25]. Finally, some rare outcomes (e.g., brachial plexus palsy, perinatal death) were too infrequent to detect differences. In summary, while findings are consistent across studies, the evidence for some endpoints is graded low/very low quality, and caution is warranted in interpretation.

### Clinical implications

The consistent finding of fewer shoulder dystocias with induction has important clinical implications. Shoulder dystocia can cause severe neonatal injuries and maternal trauma; this review suggests that planning delivery around 38 weeks can substantially reduce that risk. Clinicians should counsel patients with suspected LGA fetuses about induction around 38 weeks, highlighting that it increases the likelihood of an unassisted vaginal birth, reduces the need for caesarean delivery, and there is no clear evidence that it elevates neonatal risk. Induction is also associated with slightly lower birth weights, which may contribute to the reduction in delivery-related trauma but might also indicate trade-offs such as the need for additional postnatal care. The second notable trade-off is a higher likelihood of neonatal phototherapy following induction, likely reflecting earlier gestational age at delivery, which may lead to increased hospital stay and heightened parental concern, even in the absence of more serious complications. These benefits must be balanced against the known risks of induction (medicalization of care, longer labour, patient preference, hospital stay). However, this meta-analysis data indicate that the trade-off may now be considered more favourably by patients and healthcare professionals. Given that current guidelines (e.g., ACOG, RCOG) do not yet recommend induction for LGA, shared decision-making is key: providers should inform women that recent evidence, including the present review, shows induction can reduce the risk of shoulder dystocia, although some neonatal trade-offs, such as increased phototherapy, may occur. Future guideline revisions (such as by NICE or professional bodies) may incorporate these findings to update recommendations on managing suspected macrosomia.

## Conclusion

This meta-analysis demonstrates that induction of labour around 38 weeks for suspected large‐for‐gestational‐age fetuses significantly reduces shoulder dystocia and related birth injury and also lowers the risk of caesarean delivery, without adversely affecting other maternal outcomes. These benefits must be weighed against a significant increase in the need for neonatal phototherapy. While the data suggest a reduction in birth fractures, current evidence remains insufficient to determine the effect on very rare outcomes such as brachial plexus palsy or perinatal mortality. These findings provide moderate-certainty evidence in favour of offering or recommending early‐term induction as a management strategy for suspected macrosomia and support revisiting clinical guidelines to incorporate this option.

## Supplementary Information

Supplementary Material 1. Supplementary Appendix 1. Completed PRISMA 2020 Checklist. Checklist showing where each PRISMA 2020 reporting item is addressed in the manuscript (by page, figure, or table).

Supplementary Material 2. Table S1. Detailed GRADE evidence profile. For every analyzed outcome: number of studies/participants, pooled effect size with 95 % CI, certainty rating, and specific reasons for downgrading. Certainty categories: high, moderate, low, very low.

Supplementary Material 3. Table S2. Trustworthiness (TRACT) Checklist – Domain JudgementsTrial-level ratings across seven TRACT domains: governance, author group, plausibility of intervention, recruitment timeframe, dropout rates, baseline balance, and outcome plausibility. Overall TRACT judgement is also shown. Judgement categories: “No concerns”, “Some concerns”, and “Major concerns” based on transparency and methodological trustworthiness.

Supplementary Material 4. Figure S2. Visual abstract summary of trial design and key findings. Graphical representation of study population, comparison arms, pooled core outcomes (shoulder dystocia, caesarean birth, spontaneous vaginal birth, phototherapy need, and birthweight), and a timeline of the three included RCTs. The forest plot illustrates shoulder dystocia risk reduction with elective induction across the three trials.

Supplementary Material 5. Figure S1. ROB-2 risk-of-bias assessment. Traffic-light plot (top) and summary bar chart (bottom) for the five ROB-2 domains—D1 randomisation, D2 deviations from intended interventions, D3 missing outcome data, D4 outcome measurement, D5 selection of reported results—and the overall judgement. Green = low risk, yellow = some concerns, red = high risk.

Supplementary Material 6. Supplementary Appendix 2. Sensitivity Analyses. Forest plots incorporate data from small, unpublished trials (Libby 1998 and Tey 1995) to test the robustness of the primary findings. Inclusion of these studies did not materially change the direction or magnitude of the effect estimates.

Supplementary Material 7. Figure S3. Forest plots for secondary neonatal outcomes. Panels display common-effect (fixed) and random-effects estimates for (A) birthweight (mean difference, g), (B) need for phototherapy, (C) NICU admission and (D) low 5-min Apgar < 7 (risk ratios). Squares denote study-specific effect sizes weighted by inverse variance; horizontal lines show 95 % confidence intervals (CIs); diamonds represent pooled estimates. The vertical line marks no effect (MD = 0 g or RR = 1·0). Substantial heterogeneity was present for birthweight (I² = 92·7 %), so both models are shown; heterogeneity was negligible for the other outcomes (I² ≤ 57·5 %).

Supplementary Material 8. Figure S4. Forest plots for rare maternal and perinatal harms. Panels show common-effect and random-effects risk ratios for (A) brachial plexus injury, (B) third- or fourth-degree obstetric anal-sphincter tears, (C) birth fracture, (D) perinatal mortality, and Major postpartum haemorrhage (blood loss >1000ml). Conventions as in Figure S1: squares = study weights, lines = 95 % CIs, diamonds = pooled estimates, vertical line = no effect (RR = 1·0). Heterogeneity was low to moderate (I² ≤ 42 %) ; both models are therefore presented for all panels.

## Data Availability

All data used in this meta-analysis are from published sources. Additional data are available upon reasonable request to the corresponding author.

## Abbreviations

CI: Confidence interval
I²: Percentage of total variation across studies due to heterogeneity
MD: Mean difference
M‑H: Mantel–Haenszel
NICU: Neonatal intensive‑care unit
RR: Risk ratio
