# Supplementary material for: Elective induction versus expectant management for suspected large-for-gestational-age fetuses: a systematic review and meta-analysis
Source: BMC Pregnancy Childbirth. 2026 Feb 20;26:338. doi: 10.1186/s12884-026-08787-x (PMC13032334; doi:10.1186/s12884-026-08787-x)
Supplement: Supplementary file 1 — Supplementary Material 1. Supplementary Appendix 1. Completed PRISMA 2020 Checklist. Checklist showing where each PRISMA 2020 reporting item is addressed in the manuscript (by page, figure, or table). [file 12884_2026_8787_MOESM1_ESM.docx]

# PRISMA 2020 Checklist

For the manuscript: Elective Induction Versus Expectant Management for Suspected Large-for-Gestational-Age Fetuses: A Systematic Review and Meta-Analysis

| Section & Topic | Item # | Checklist Item | Location in Manuscript |
| --- | --- | --- | --- |
| TITLE | 1 | Identify the report as a systematic review | Title page |
| ABSTRACT | 2 | Provide a structured summary | Structured Abstract |
| INTRODUCTION | 3 | Rationale for the review | Introduction (pp. 2–3) |
| INTRODUCTION | 4 | Explicit statement of questions | End of Introduction |
| METHODS | 5 | Eligibility criteria | Search strategy (p. 4) |
| METHODS | 6 | Information sources | Search strategy (p. 4) |
| METHODS | 7 | Search strategy details | Supplementary Appendix 1 |
| METHODS | 8 | Selection process | Methods and PRISMA flowchart (Fig. 1) |
| METHODS | 9 | Data collection process | Methods (p. 5) |
| METHODS | 10a | Outcomes defined | Outcomes section (p. 5) |
| METHODS | 10b | Other data items | Participant characteristics, Table 1 |
| METHODS | 11 | Risk of bias assessment | Methods (p. 5) |
| METHODS | 12 | Effect measures specified | Methods (p. 5) |
| METHODS | 13a–f | Synthesis methods and analyses | Methods (p. 5–6) |
| METHODS | 14 | Reporting bias assessment | Methods (p. 6) |
| METHODS | 15 | Certainty assessment (e.g., GRADE) | Methods (p. 6), Table S1 |
| RESULTS | 16a–b | Study selection and exclusions | Results (p. 7), Fig. 1 |
| RESULTS | 17 | Study characteristics | Table 1 |
| RESULTS | 18 | Risk of bias in studies | Results (p. 10), Fig. S1 |
| RESULTS | 19 | Results of individual studies | Forest plots (Fig. 2, Supp.) |
| RESULTS | 20a–d | Synthesis results | Results (pp. 11–12) |
| RESULTS | 21 | Reporting biases | Results (p. 6) |
| RESULTS | 22 | Certainty of evidence | Summary of Findings Table |
| DISCUSSION | 23a–d | Interpretation, limitations, implications | Discussion (pp. 13–15) |
| OTHER | 24a–c | Registration and protocol | Methods (p. 3) |
| OTHER | 25 | Support | Title page |
| OTHER | 26 | Competing interests | Title page |
| OTHER | 27 | Data availability | Title page |
