# Supplementary material for: Elective induction versus expectant management for suspected large-for-gestational-age fetuses: a systematic review and meta-analysis
Source: BMC Pregnancy Childbirth. 2026 Feb 20;26:338. doi: 10.1186/s12884-026-08787-x (PMC13032334; doi:10.1186/s12884-026-08787-x)
Supplement: Supplementary file 2 — Supplementary Material 2. Table S1. Detailed GRADE evidence profile. For every analyzed outcome: number of studies/participants, pooled effect size with 95 % CI, certainty rating, and specific reasons for downgrading. Certainty categories: high, moderate, low, very low. [file 12884_2026_8787_MOESM2_ESM.docx]

# Trustworthiness (TRACT) Checklist – Domain Judgements

| Trial | Domain 1: Governance | Domain 2: Author group | Domain 3: Plausibility of intervention | Domain 4: Timeframe / recruitment pace | Domain 5: Drop‑out rates | Domain 6: Baseline balance | Domain 7: Outcome plausibility | Overall TRACT |
| --- | --- | --- | --- | --- | --- | --- | --- | --- |
| Boulvain 2015 – Induction vs expectant (LGA) | Some concerns | No concerns | No concerns | No concerns | No concerns | No concerns | No concerns | Some concerns |
| Gonen 1997 – Induction vs expectant (macrosomia) | Some concerns | No concerns | No concerns | Some concerns | No concerns | Some concerns | No concerns | Some concerns |
| Gardosi 2025 – “Big Baby” trial | No concerns | No concerns | No concerns | No concerns | No concerns | No concerns | No concerns | No concerns |

## Judgement categories

No concerns – Adequate information; no evident methodological or reporting issues.
Some concerns – Minor or moderate issues that introduce some uncertainty.
Major concerns – Serious flaws or missing information likely to bias results.
