# Supplementary figures and images for: Elective induction versus expectant management for suspected large-for-gestational-age fetuses: a systematic review and meta-analysis
Source: BMC Pregnancy Childbirth. 2026 Feb 20;26:338. doi: 10.1186/s12884-026-08787-x (PMC13032334; doi:10.1186/s12884-026-08787-x)

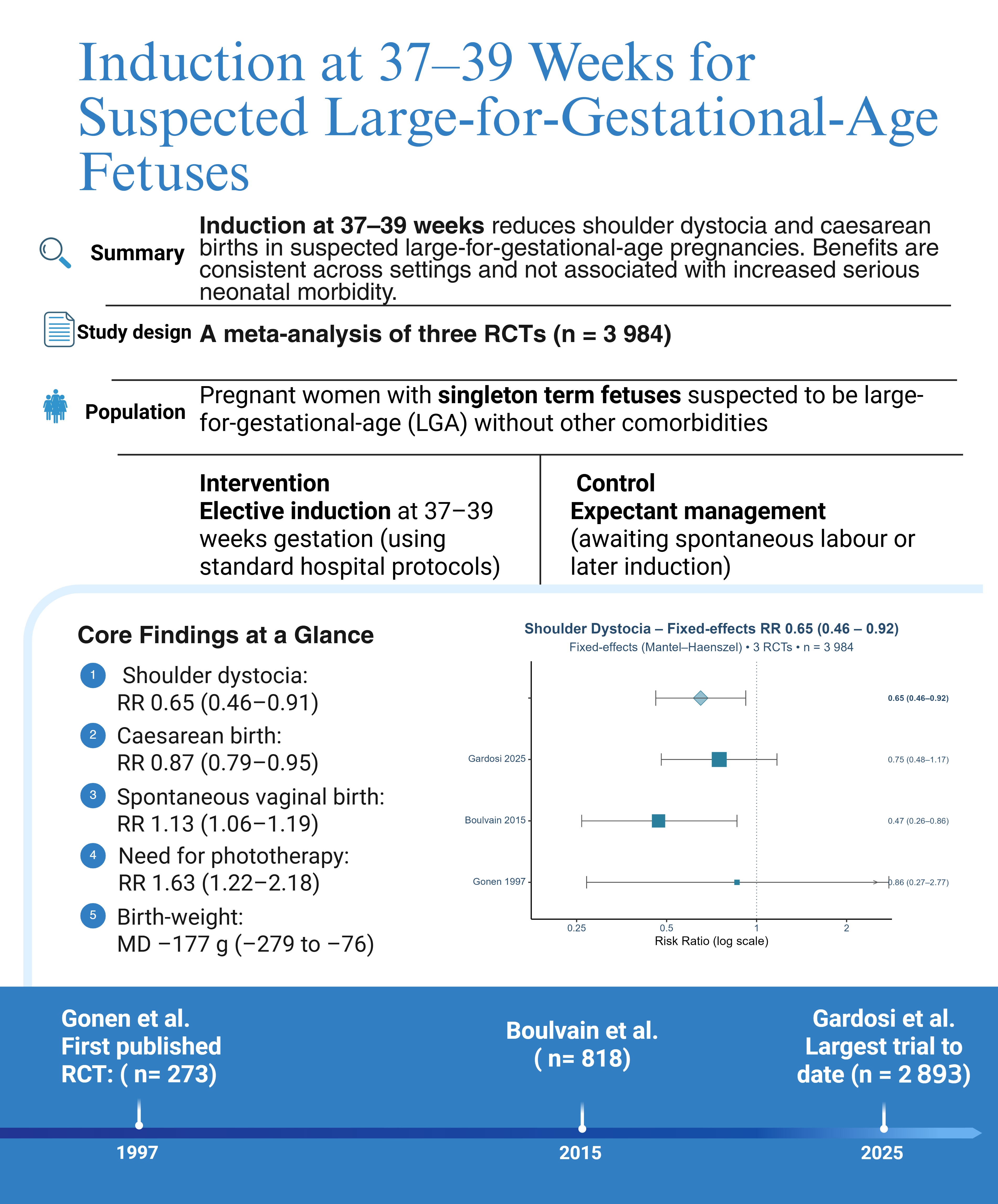

Supplement: Supplementary file 3 — Supplementary Material 3. Table S2. Trustworthiness (TRACT) Checklist – Domain JudgementsTrial-level ratings across seven TRACT domains: governance, author group, plausibility of intervention, recruitment timeframe, dropout rates, baseline balance, and outcome plausibility. Overall TRACT judgement is also shown. Judgement categories: “No concerns”, “Some concerns”, and “Major concerns” based on transparency and methodological trustworthiness. [file 12884_2026_8787_MOESM3_ESM.png]

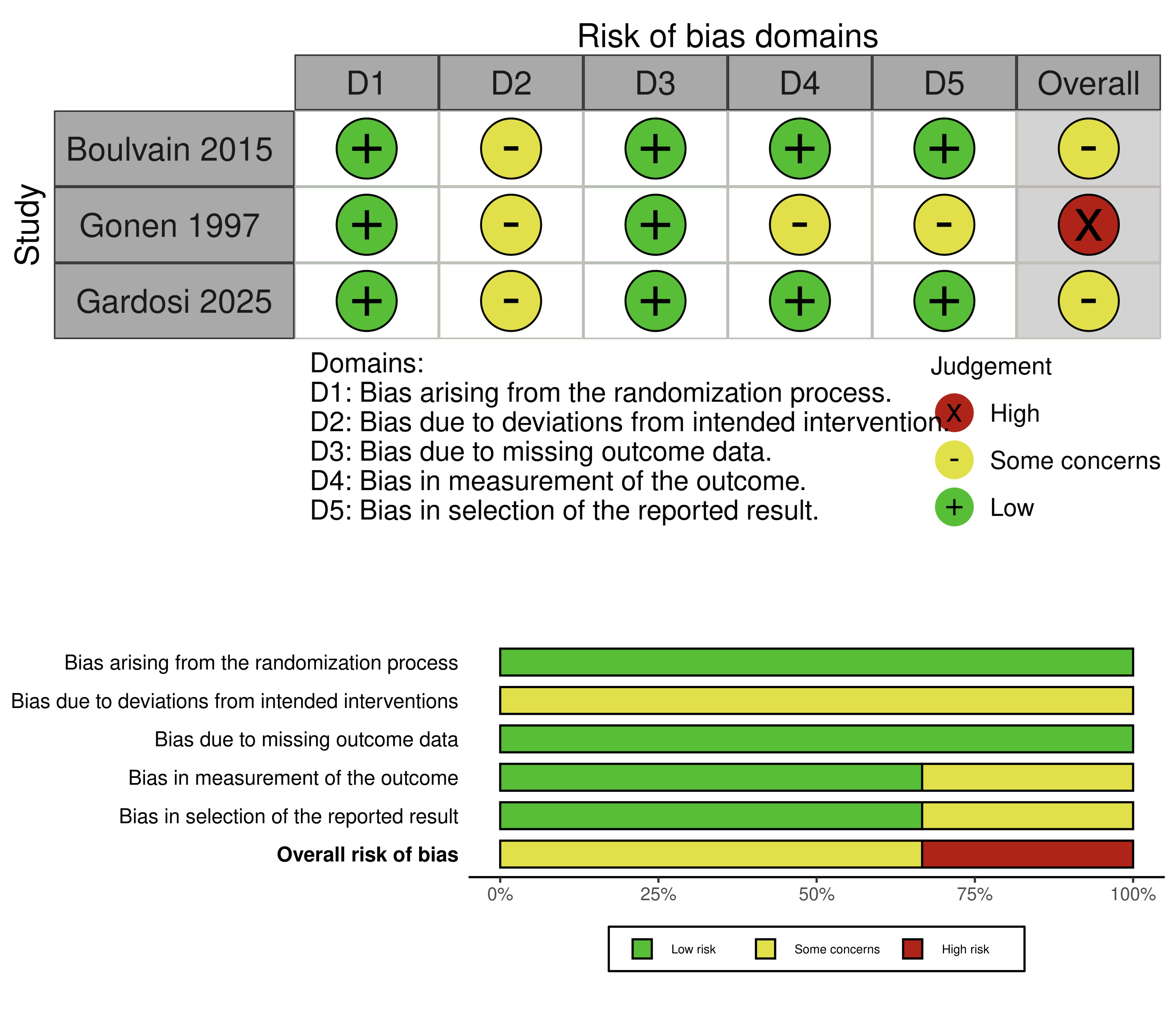

Supplement: Supplementary file 5 — Supplementary Material 5. Figure S1. ROB-2 risk-of-bias assessment. Traffic-light plot (top) and summary bar chart (bottom) for the five ROB-2 domains—D1 randomisation, D2 deviations from intended interventions, D3 missing outcome data, D4 outcome measurement, D5 selection of reported results—and the overall judgement. Green = low risk, yellow = some concerns, red = high risk. [file 12884_2026_8787_MOESM5_ESM.jpg]

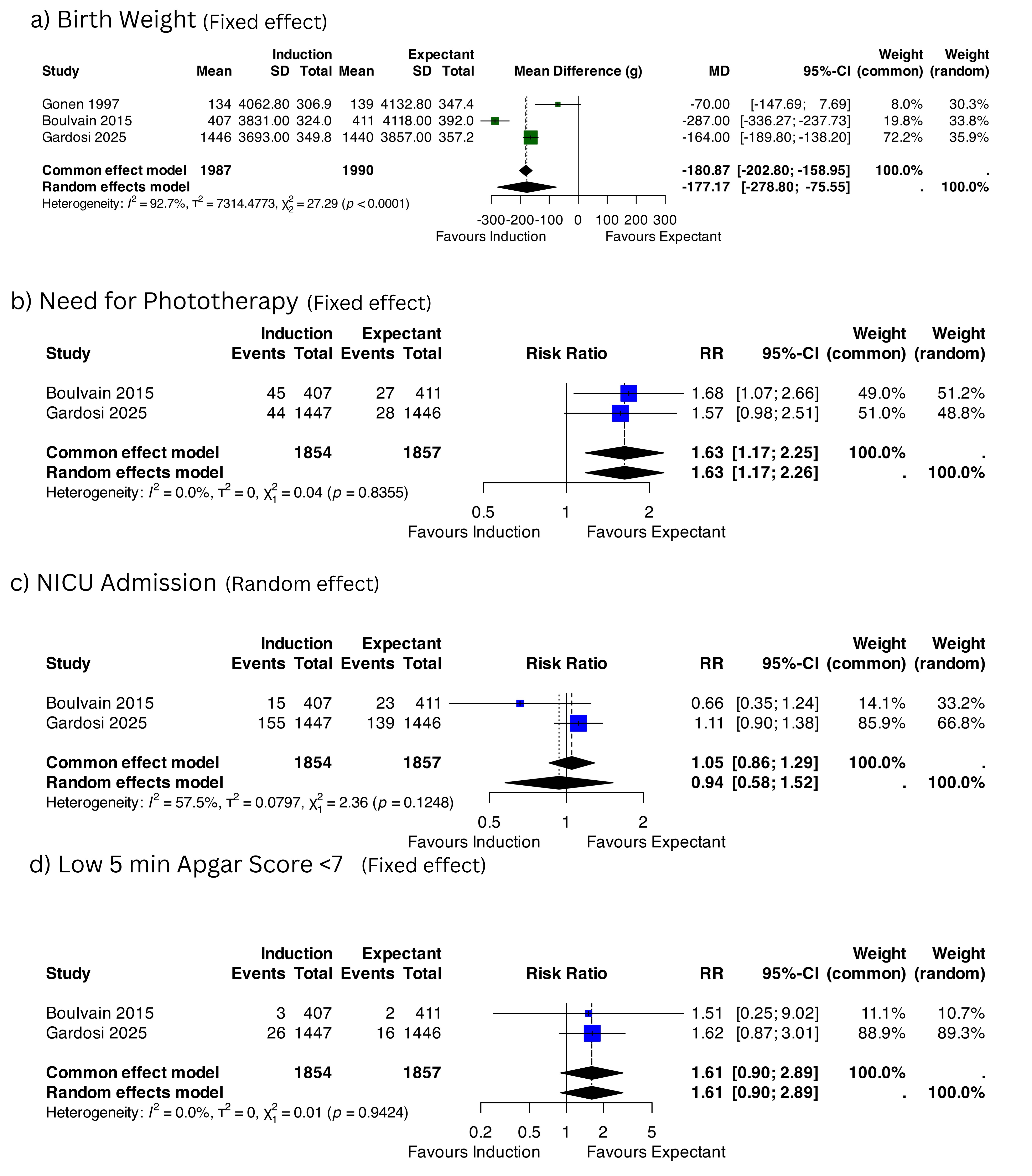

Supplement: Supplementary file 7 — Supplementary Material 7. Figure S3. Forest plots for secondary neonatal outcomes. Panels display common-effect (fixed) and random-effects estimates for (A) birthweight (mean difference, g), (B) need for phototherapy, (C) NICU admission and (D) low 5-min Apgar < 7 (risk ratios). Squares denote study-specific effect sizes weighted by inverse variance; horizontal lines show 95 % confidence intervals (CIs); diamonds represent pooled estimates. The vertical line marks no effect (MD = 0 g or RR = 1·0). Substantial heterogeneity was present for birthweight (I² = 92·7 %), so both models are shown; heterogeneity was negligible for the other outcomes (I² ≤ 57·5 %). [file 12884_2026_8787_MOESM7_ESM.png]

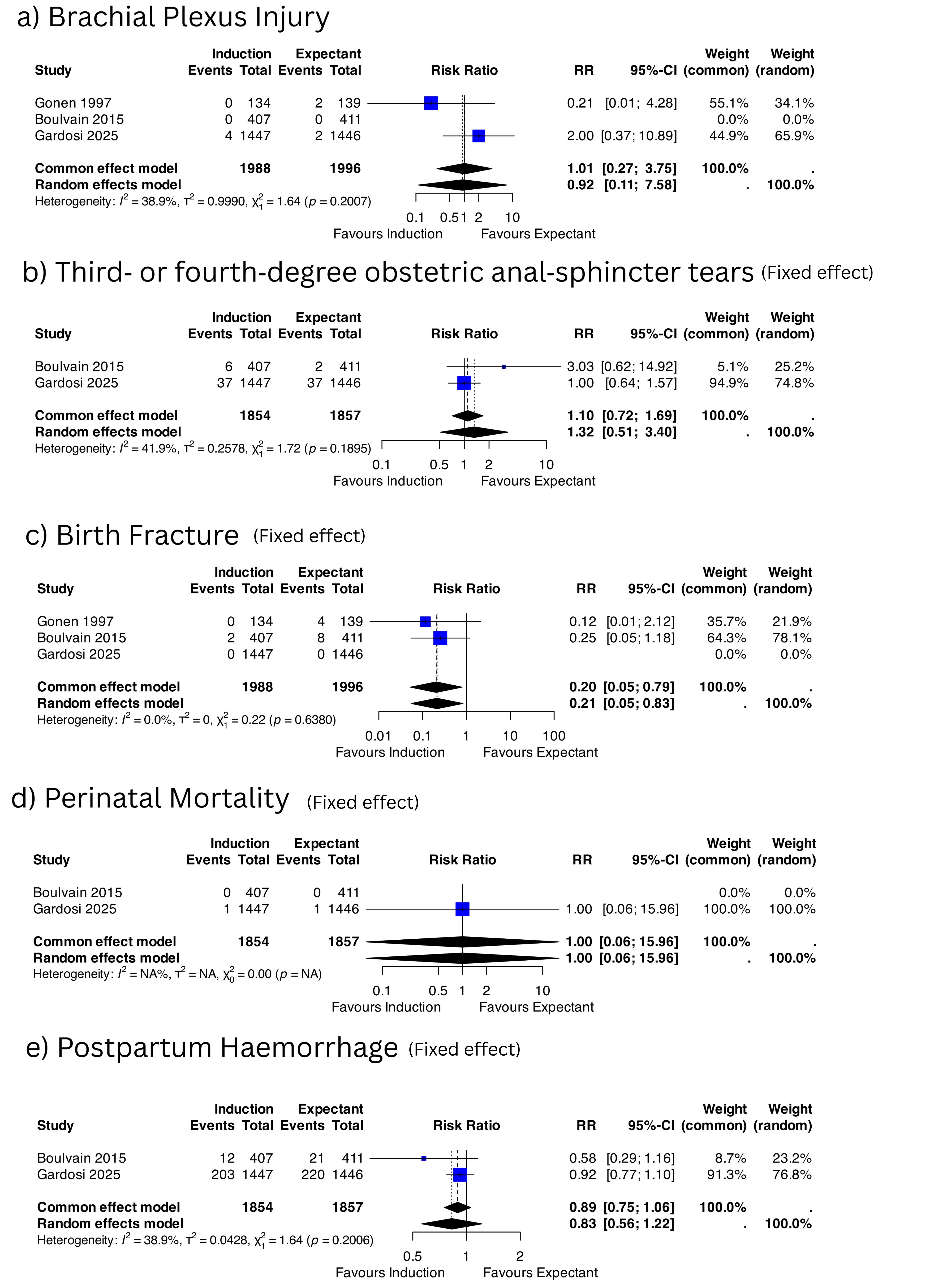

Supplement: Supplementary file 8 — Supplementary Material 8. Figure S4. Forest plots for rare maternal and perinatal harms. Panels show common-effect and random-effects risk ratios for (A) brachial plexus injury, (B) third- or fourth-degree obstetric anal-sphincter tears, (C) birth fracture, (D) perinatal mortality, and Major postpartum haemorrhage (blood loss >1000ml). Conventions as in Figure S1: squares = study weights, lines = 95 % CIs, diamonds = pooled estimates, vertical line = no effect (RR = 1·0). Heterogeneity was low to moderate (I² ≤ 42 %) ; both models are therefore presented for all panels. [file 12884_2026_8787_MOESM8_ESM.png]
