# Supplementary material for: Elective induction versus expectant management for suspected large-for-gestational-age fetuses: a systematic review and meta-analysis
Source: BMC Pregnancy Childbirth. 2026 Feb 20;26:338. doi: 10.1186/s12884-026-08787-x (PMC13032334; doi:10.1186/s12884-026-08787-x)
Supplement: Supplementary file 4 — Supplementary Material 4. Figure S2. Visual abstract summary of trial design and key findings. Graphical representation of study population, comparison arms, pooled core outcomes (shoulder dystocia, caesarean birth, spontaneous vaginal birth, phototherapy need, and birthweight), and a timeline of the three included RCTs. The forest plot illustrates shoulder dystocia risk reduction with elective induction across the three trials. [file 12884_2026_8787_MOESM4_ESM.pdf]

Study or Subgroup	Induction Events	Induction Total	Expectant Events	Expectant Total	Weight	Risk Ratio M-H, Fixed, 95% CI
Boulvain 2015	15	407	32	411	37.6%	0.47 [0.26, 0.86]
Gardosi 2025	33	1445	44	1439	52.1%	0.75 [0.48, 1.17]
Gonen 1997	5	134	6	139	7.0%	0.86 [0.27, 2.77]
Libby 1998	0	30	0	29		Not estimable
Tey 1995	4	19	3	21	3.4%	1.47 [0.38, 5.75]
**Total (95% CI)**		**2035**		**2039**	**100.0%**	**0.68 [0.49, 0.94]**
Total events	57		85			

Heterogeneity: Chi² = 2.99, df = 3 (P = 0.39); I² = 0%
Test for overall effect: Z = 2.33 (P = 0.02)

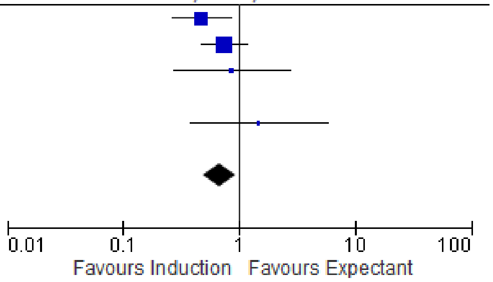

Figure 1 Caesarean Section

Study or Subgroup	Induction Events	Induction Total	Expectant Events	Expectant Total	Weight	Risk Ratio M-H, Fixed, 95% CI
Boulvain 2015	114	407	130	411	19.8%	0.89 [0.72, 1.09]
Gardosi 2025	411	1447	477	1446	73.2%	0.86 [0.77, 0.96]
Gonen 1997	26	134	30	139	4.5%	0.90 [0.56, 1.44]
Libby 1998	11	30	8	29	1.2%	1.33 [0.63, 2.83]
Tey 1995	6	19	8	21	1.2%	0.83 [0.35, 1.95]
**Total (95% CI)**		**2037**		**2046**	**100.0%**	**0.87 [0.79, 0.96]**
Total events	568		653			

Heterogeneity: Chi² = 1.30, df = 4 (P = 0.86); I² = 0%
Test for overall effect: Z = 2.83 (P = 0.005)

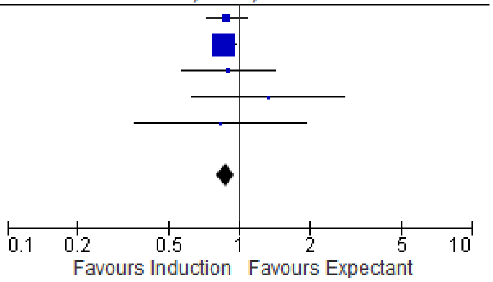

Figure 2 Shoulder Dystocia

Study or Subgroup	Induction Events	Induction Total	Expectant Events	Expectant Total	Weight	Risk Ratio M-H, Fixed, 95% CI	Year
Tey 1995	0	19	0	21		Not estimable	1995
Gonen 1997	0	134	2	139	55.1%	0.21 [0.01, 4.28]	1997
Libby 1998	0	30	0	29		Not estimable	1998
Boulvain 2015	0	407	0	411		Not estimable	2015
Gardosi 2025	4	1447	2	1446	44.9%	2.00 [0.37, 10.89]	2025
**Total (95% CI)**		**2037**		**2046**	**100.0%**	**1.01 [0.27, 3.75]**	
Total events	4		4				

Heterogeneity: Chi² = 1.67, df = 1 (P = 0.20); I² = 40%
Test for overall effect: Z = 0.02 (P = 0.99)

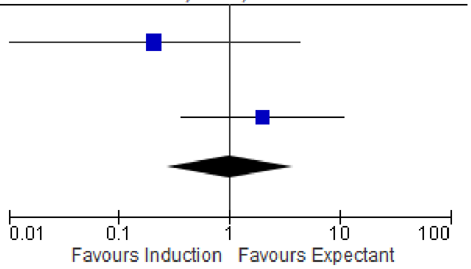

Figure 3 Brachial Plexus Injury

Study or Subgroup	Induction Events	Induction Total	Expectant Events	Expectant Total	Weight	Risk Ratio M-H, Fixed, 95% CI
Tey 1995	13	19	13	21	1.2%	1.11 [0.70, 1.74]
Libby 1998	13	30	16	29	1.6%	0.79 [0.46, 1.33]
Gonen 1997	91	134	91	139	8.6%	1.04 [0.88, 1.23]
Boulvain 2015	239	407	212	411	20.4%	1.14 [1.01, 1.29]
Gardosi 2025	799	1447	704	1446	68.2%	1.13 [1.06, 1.22]
**Total (95% CI)**		**2037**		**2046**	**100.0%**	**1.12 [1.06, 1.19]**
Total events	1155		1036			

Heterogeneity: Chi² = 2.76, df = 4 (P = 0.60); I² = 0%
Test for overall effect: Z = 3.92 (P < 0.0001)

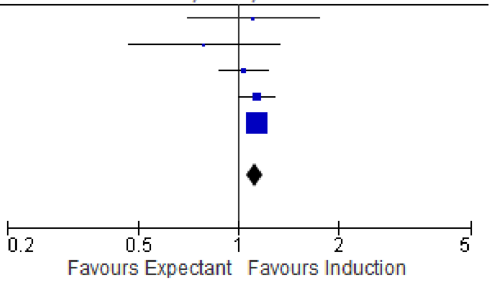

Figure 4 Spontaneous Delivery

Study or Subgroup	Induction Mean	Induction SD	Induction Total	Expectant Mean	Expectant SD	Expectant Total	Weight	Mean Difference IV, Random, 95% CI	Year
Tey 1995	4,250	317	19	4,253	338	21	10.6%	-3.00 [-206.02, 200.02]	1995
Gonen 1997	4,062.8	306.9	134	4,132.8	347.4	139	21.4%	-70.00 [-147.69, 7.69]	1997
Libby 1998	3,705	148.15	30	4,000	251.85	29	18.6%	-295.00 [-400.89, -189.11]	1998
Boulvain 2015	3,831	324	407	4,118	392	411	23.9%	-287.00 [-336.27, -237.73]	2015
Gardosi 2025	3,693	349.8	1446	3,857	357.2	1440	25.4%	-164.00 [-189.80, -138.20]	2025
**Total (95% CI)**			**2036**			**2040**	**100.0%**	**-180.58 [-266.76, -94.40]**	

Heterogeneity: Tau² = 7450.11; Chi² = 34.65, df = 4 (P < 0.00001); I² = 88%
Test for overall effect: Z = 4.11 (P < 0.0001)

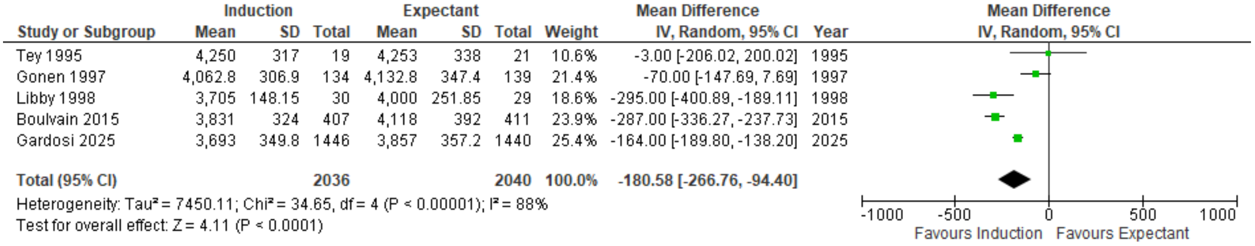

Figure 5 Mean Birth Weight

Study or Subgroup	Induction Events	Induction Total	Expectant Events	Expectant Total	Weight	Risk Ratio M-H, Fixed, 95% CI	Year
Tey 1995	0	19	0	21		Not estimable	1995
Gonen 1997	0	134	4	139	35.7%	0.12 [0.01, 2.12]	1997
Libby 1998	0	30	0	29		Not estimable	1998
Boulvain 2015	2	407	8	411	64.3%	0.25 [0.05, 1.18]	2015
Gardosi 2025	0	1447	0	1446		Not estimable	2025
**Total (95% CI)**		**2037**		**2046**	**100.0%**	**0.20 [0.05, 0.79]**	
Total events	2		12				

Heterogeneity: Chi² = 0.22, df = 1 (P = 0.64); I² = 0%
Test for overall effect: Z = 2.30 (P = 0.02)

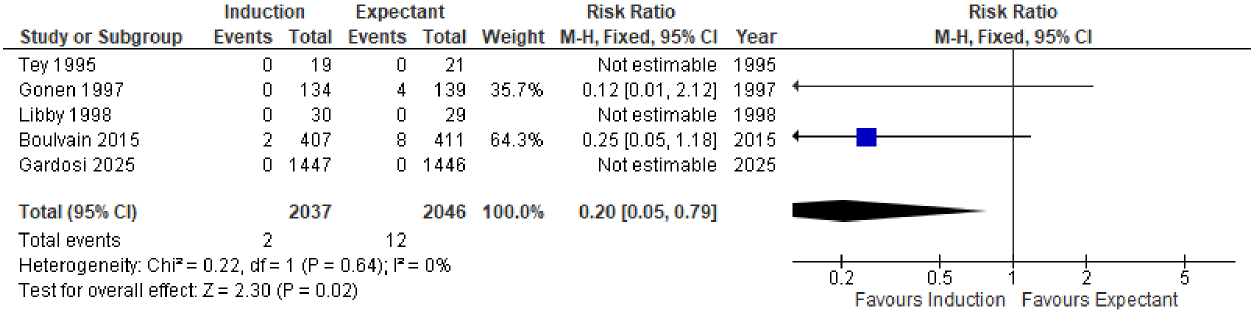

Figure 6 Fractures

Study or Subgroup	Induction Events	Induction Total	Expectant Events	Expectant Total	Weight	Risk Ratio M-H, Fixed, 95% CI
Boulvain 2015	3	407	2	411	11.1%	1.51 [0.25, 9.02]
Gardosi 2025	26	1447	16	1446	88.9%	1.62 [0.87, 3.01]
Tey 1995	0	19	0	21		Not estimable
**Total (95% CI)**		**1873**		**1878**	**100.0%**	**1.61 [0.90, 2.89]**
Total events	29		18			

Heterogeneity: Chi² = 0.01, df = 1 (P = 0.94); I² = 0%
Test for overall effect: Z = 1.60 (P = 0.11)

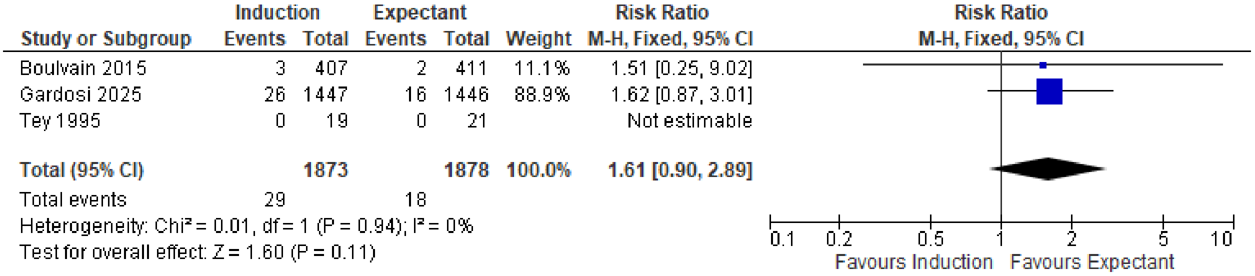

Figure 7 Low Apgar Score

Study or Subgroup	Induction Events	Induction Total	Expectant Events	Expectant Total	Weight	Risk Ratio M-H, Fixed, 95% CI	Year
Tey 1995	0	19	0	21		Not estimable	1995
Boulvain 2015	6	407	2	411	5.1%	3.03 [0.62, 14.92]	2015
Gardosi 2025	37	1447	37	1446	94.9%	1.00 [0.64, 1.57]	2025
**Total (95% CI)**		**1873**		**1878**	**100.0%**	**1.10 [0.72, 1.69]**	
Total events	43		39				

Heterogeneity: Chi² = 1.73, df = 1 (P = 0.19); I² = 42%
Test for overall effect: Z = 0.45 (P = 0.65)

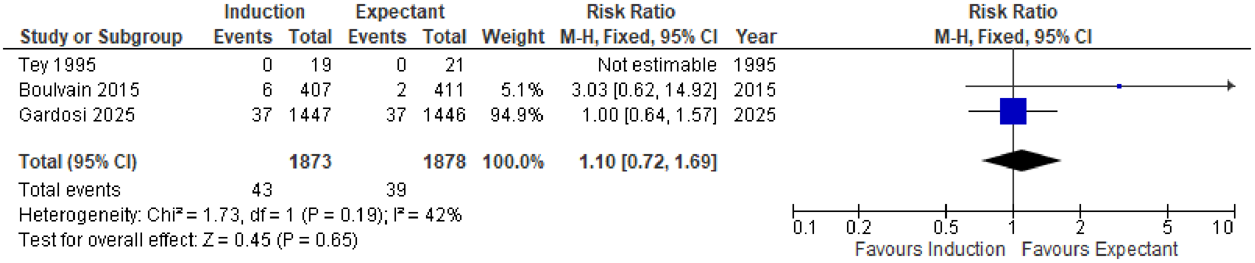

Figure 8 Third- and Fourth-Degree Anal Sphincter Tear
