# Supplementary material for: Elective induction versus expectant management for suspected large-for-gestational-age fetuses: a systematic review and meta-analysis
Source: BMC Pregnancy Childbirth. 2026 Feb 20;26:338. doi: 10.1186/s12884-026-08787-x (PMC13032334; doi:10.1186/s12884-026-08787-x)
Supplement: Supplementary file 6 — Supplementary Material 6. Supplementary Appendix 2. Sensitivity Analyses. Forest plots incorporate data from small, unpublished trials (Libby 1998 and Tey 1995) to test the robustness of the primary findings. Inclusion of these studies did not materially change the direction or magnitude of the effect estimates. [file 12884_2026_8787_MOESM6_ESM.docx]

# GRADE Domain Judgements – Summary Table

Downgrades: “Serious” = −1 level; “Very serious” = −2 levels. Certainty categories: High, Moderate, Low, Very low.

| Outcome | Participants (studies) | Risk of bias | Inconsistency | Indirectness | Imprecision | Publication bias | Certainty | Effect (95 % CI) |
| --- | --- | --- | --- | --- | --- | --- | --- | --- |
| Shoulder dystocia | 3 975 (3) | Some concerns | Not serious | Not serious | Not serious | Undetected | Moderate | RR 0.65 (0.46–0.91) |
| Brachial‑plexus injury | 3 984 (3) | Some concerns | Not serious | Not serious | Serious | Undetected | Low | RR 1.01 (0.27–3.75) |
| Birth fracture | 3 984 (3) | Some concerns | Not serious | Not serious | Serious | Undetected | Low | RR 0.20 (0.05–0.79) |
| Caesarean section | 3 988 (3) | Some concerns | Not serious | Not serious | Not serious | Undetected | Moderate | RR 0.87 (0.79–0.95) |
| Instrumental vaginal birth | 3 984 (3) | Some concerns | Not serious | Not serious | Serious | Undetected | Low | RR 0.89 (0.78–1.03) |
| Spontaneous vaginal birth | 3 984 (3) | Some concerns | Not serious | Not serious | Not serious | Undetected | Moderate | RR 1.13 (1.06–1.19) |
| 3rd/4th‑degree perineal tear | 3 711 (2) | Some concerns | Not serious | Not serious | Serious | Undetected | Low | RR 1.10 (0.72–1.69) |
| Birth‑weight (g) | 3 977 (3) | Some concerns | Serious | Not serious | Not serious | Undetected | Low | MD −177 g (−279 to −76) |
| Need for phototherapy | 3 701 (2) | Some concerns | Not serious | Not serious | Not serious | Undetected | Moderate | RR 1.63 (1.17–2.25) |
| NICU admission | 3 701 (2) | Some concerns | Serious | Not serious | Serious | Undetected | Low | RR 0.94 (0.58–1.52) |
| Apgar <7 at 5 min | 3 711 (2) | Some concerns | Not serious | Not serious | Serious | Undetected | Low | RR 1.61 (0.90–2.89) |
| Major Postpartum Haemorrhage | 3 711 (2) | Some concerns | Not serious | Not serious | Serious | Undetected | Low | RR 0.89 (0.75–1.06) |
| Perinatal mortality | 3 741 (2) | Some concerns | Not applicable | Not serious | Very serious | Undetected | Very low | RR 1.00 (0.06–15.96) |

“Not applicable” for inconsistency when only one study contributed an event.
